# How can health systems approach reducing health inequalities? An in-depth qualitative case study in the UK

**DOI:** 10.1186/s12889-024-19531-5

**Published:** 2024-08-10

**Authors:** Charlotte Parbery-Clark, Lorraine McSweeney, Joanne Lally, Sarah Sowden

**Affiliations:** 1 Faculty of Medical Sciences, Public Health Registrar, Population Health Sciences Institute, Newcastle University, Newcastle Upon Tyne, UK; 2 Post-Doctoral Research Associate, Faculty of Medical Sciences, Population Health Sciences Institute, Newcastle University, Newcastle Upon Tyne, UK; 3 Senior Research Methodologist & Public Involvement Lead, Faculty of Medical Sciences, Population Health Sciences Institute, Newcastle University, Newcastle Upon Tyne, UK; 4 Senior Clinical Lecturer &, Faculty of Medical Sciences, Honorary Consultant in Public Health, Population Health Sciences Institute, Newcastle University, Newcastle Upon Tyne, UK

**Keywords:** Health inequalities, Complex whole systems approach, In-depth qualitative case study

## Abstract

**Background:**

Addressing socioeconomic inequalities in health and healthcare, and reducing avoidable hospital admissions requires integrated strategy and complex intervention across health systems. However, the understanding of how to create effective systems to reduce socio-economic inequalities in health and healthcare is limited. The aim was to explore and develop a system’s level understanding of how local areas address health inequalities with a focus on avoidable emergency admissions.

**Methods:**

In-depth case study using qualitative investigation (documentary analysis and key informant interviews) in an urban UK local authority. Interviewees were identified using snowball sampling. Documents were retrieved via key informants and web searches of relevant organisations. Interviews and documents were analysed independently based on a thematic analysis approach.

**Results:**

Interviews (*n* = 14) with wide representation from local authority (*n* = 8), NHS (*n* = 5) and voluntary, community and social enterprise (VCSE) sector (*n* = 1) with 75 documents (including from NHS, local authority, VCSE) were included. Cross-referenced themes were understanding the local context, facilitators of how to tackle health inequalities: the assets, and emerging risks and concerns. Addressing health inequalities in avoidable admissions per se was not often explicitly linked by either the interviews or documents and is not yet embedded into practice. However, a strong coherent strategic integrated population health management plan with a system’s approach to reducing health inequalities was evident as was collective action and involving people, with links to a “strong third sector”. Challenges reported include structural barriers and threats, the analysis and accessibility of data as well as ongoing pressures on the health and care system.

**Conclusion:**

We provide an in-depth exploration of how a local area is working to address health and care inequalities. Key elements of this system’s working include fostering strategic coherence, cross-agency working, and community-asset based approaches. Areas requiring action included data sharing challenges across organisations and analytical capacity to assist endeavours to reduce health and care inequalities. Other areas were around the resilience of the system including the recruitment and retention of the workforce. More action is required to embed reducing health inequalities in avoidable admissions explicitly in local areas with inaction risking widening the health gap.

**Supplementary Information:**

The online version contains supplementary material available at 10.1186/s12889-024-19531-5.

## Highlights

• Reducing health inequalities in avoidable hospital admissions is yet to be explicitly linked in practice and is an important area to address.

• Understanding the local context helps to identify existing assets and threats including the leverage points for action.

• Requiring action includes building the resilience of our complex systems by addressing structural barriers and threats as well as supporting the workforce (training and wellbeing with improved retention and recruitment) in addition to the analysis and accessibility of data across the system.

**Supplementary Information:**

The online version contains supplementary material available at 10.1186/s12889-024-19531-5.

## Introduction

The health of our population is determined by the complex interaction of several factors which are either non-modifiable (such as age, genetics) or modifiable (such as the environment, social, economic conditions in which we live, our behaviours as well as our access to healthcare and its quality) [1]. Health inequalities are the avoidable and unfair systematic differences in health and healthcare across different population groups explained by the differences in distribution of power, wealth and resources which drive the conditions of daily life [2, 3]. Essentially, health inequalities arise due to the systematic differences of the factors that influence our health. To effectively deal with most public health challenges, including reducing health inequalities and improving population health, broader integrated approaches [4] and an emphasis on systems is required [5, 6]*.* A system is defined as ‘the set of actors, activities, and settings that are directly or indirectly perceived to have influence in or be affected by a given problem situation’ (p.198) [7]. In this case, the ‘given problem situation' is reducing health inequalities with a focus on avoidable admissions. Therefore, we must consider health systems, which are the organisations, resources and people aiming to improve or maintain health [8, 9] of which health services provision is an aspect. In this study, the system considers NHS bodies, Integrated Care Systems, Local Authority departments, and the voluntary and community sector in a UK region.

A plethora of theories [10], recommended policies [3, 11–13], frameworks [1, 14, 15], and tools [16] exist to help understand the existence of health inequalities as well as provide suggestions for improvement. However, it is reported that healthcare leaders feel under-skilled to reduce health inequalities [17]. A lack of clarity exists on how to achieve a system’s multi-agency coherence to reduce health inequalities systematically [17, 18]. This is despite some countries having legal obligations to have a regard to the need to attend to health and healthcare inequalities. For example, the Health and Social Care Act 2012 [19], in England, mandated Clinical Commissioning Groups (CCGs), now transferred to Integrated Care Boards (ICBs) [20], to ‘have a regard to the need to reduce inequalities between patients with respect to their ability to access health services, and reduce inequalities between patients with respect to the outcomes achieved for them by the provision of health services’. The wider determinants of health must also be considered. For example, local areas have a mandatory requirement to have a joint strategic needs assessment (JSNA) and joint health and wellbeing strategy (JHWS) whose purpose is to ‘improve the health and wellbeing of the local community and reduce inequalities for all ages' [21] This includes addressing the wider determinants of health [21]. Furthermore, the hospital care costs to the NHS associated with socioeconomic inequalities has been previously reported at £4.8 billion a year due to excess hospitalisations [22]. Avoidable emergency admissions are admissions into hospital that are considered to be preventable with high-quality ambulatory care [23]. Both ambulatory care sensitive conditions (where effective personalised care based in the community can aid the prevention of needing an admission) and urgent care sensitive conditions (where a system on the whole should be able to treat and manage without an admission) are considered within this definition [24] (encompassing more than 100 International Classification of Diseases (ICD) codes). The disease burden sits disproportionately with our most disadvantaged communities, therefore highlighting the importance of addressing inequalities in hospital pressures in a concerted manner [25, 26].

Research examining one component of an intervention, or even one part of the system, [27] or which uses specific research techniques to control for the system’s context [28] are considered as having limited use for identifying the key ingredients to achieve better population health and wellbeing [5, 28]. Instead, systems thinking considers how the system’s components and sub-components interconnect and interrelate within and between each other (and indeed other systems) to gain an understanding of the mechanisms by which things work [29, 30]. Complex interventions or work programmes may perform differently in varying contexts and through different mechanisms, and therefore cannot simply be replicated from one context to another to automatically achieve the same outcomes. Ensuring that research into systems and systems thinking considers real-world context, such as where individuals live, where policies are created and interventions are delivered, is vital [5]. How the context and implementation of complex or even simple interventions interact is viewed as becoming increasingly important [31, 32]. Case study research methodology is founded on the ‘in-depth exploration of complex phenomena in their natural, or ‘real-life’, settings’ (p.2) [33]. Case study approaches can deepen the understanding of complexity addressing the ‘how’, ‘what’ and ‘why’ questions in a real-life context [34]. Researchers have highlighted the importance of engaging more deeply with case-based study methodology [31, 33]. Previous case study research has shown promise [35] which we build on by exploring a systems lens to consider the local area’s context [16] within which the work is implemented. By using case-study methodology, our study aimed to explore and develop an in-depth understanding of how a local area addresses health inequalities, with a focus on avoidable hospital admissions. As part of this, systems processes were included.

## Methods

### Study design

This in-depth case study is part of an ongoing larger multiple (collective [36]) case study approach. An instrumental approach [34] was taken allowing an in-depth investigation of an issue, event or phenomenon, in its natural real-life context; referred to as a ‘naturalistic’ design [34]. Ethics approval was obtained by Newcastle University’s Ethics Committee (ref 13633/2020).

### Study selection

This case study, alongside the other three cases, was purposively [36] chosen considering overall deprivation level of the area (Indices of Multiple Deprivation (IMD) [37]), their urban/rural location, differing geographical spread across the UK (highlighted in patient and public feedback and important for considering the North/South health divide [38]), and a pragmatic judgement of likely ability to achieve the depth of insight required [39]. In this paper, we report the findings from one of the case studies, an urban local authority in the Northern region of the UK with high levels of socioeconomic disadvantage. This area was chosen for this in-depth case analysis due to high-level of need, and prior to the COVID-19 pandemic (2009-2018) had experienced a trend towards reducing socioeconomic inequalities in avoidable hospital admission rates between neighbourhoods within the local area [40]. Thereby this case study represents an ‘unusual’ case [41] to facilitate learning regarding what is reported and considered to be the key elements required to reduce health inequalities, including inequalities in avoidable admissions, in a local area.

### Semi-structured interviews

The key informants were identified iteratively through the documentary analysis and in consultation with the research advisory group. Initially board level committee members (including lay, managerial, and clinical members) within relevant local organisations were purposively identified. These individuals were systems leaders charged with the remit of tackling health inequalities and therefore well placed to identify both key personnel and documents. Snowball sampling [42] was undertaken thereafter whereby interviewees helped to identify additional key informants within the local system who were working on health inequalities, including avoidable emergency admissions, at a systems level. Interview questions were based on an iteratively developed topic guide (supplementary data 1), informed from previous work’s findings [43] and the research advisory network’s input. A study information sheet was emailed to perspective interviewees, and participants were asked to complete an e-consent form using Microsoft Forms [42]. Each interviewee was interviewed by either L.M. or C.P.-C. using the online platforms Zoom or Teams, and lasted up to one hour. Participants were informed of interviewers’ role, workplace as well as purpose of the study. Interviewees were asked a range of questions including any work relating to reducing health inequalities, particularly avoidable emergency admissions, within the last 5 years. Brief notes were taken, and the interviews were recorded, transcribed verbatim and anonymised.

### Documentary analysis

The documentary analysis followed the READ approach [44]. Any documents from the relevant local/regional area with sections addressing health inequalities and/or avoidable emergency admissions, either explicitly stated or implicitly inferred, were included. A list of core documents was chosen, including the local Health and Wellbeing Strategy (Table 1). Subsequently, other documents were identified by snowballing from these core documents and identification by the interviewees. All document types were within scope if produced/covered a period within 5 years (2017-2022), including documents in the public domain or not as well as documents pertaining to either a regional, local and neighbourhood level. This 5-year period was a pragmatic decision in line with the interviews and considered to be a balance of legacy and relevance. Attempts were made to include the final version of each document, where possible/applicable, otherwise the most up-to-date version or version available was used.

**Table 1:** List of core documents for the documentary analysis

**Regional**
**1.** Integrated Care System (ICS) strategy
**2.** ICS strategy outlining approach to Long Term Plan (LTP)
**Local**
**3.** Health and Wellbeing Strategy
**4.** Joint Strategic Needs Assessment (JSNA)
**5.** Director of Public Health (DPH) annual report
**6.** Clinical Commissioning Group (CCG) annual report/strategic plan
**7.** Strategy addressing/outlining approach to Health Inequalities Framework
**8.** Strategy/policy outlining approach to LTP

An Excel spreadsheet data extraction tool was adapted with a priori criteria [44] to extract the data. This tool included contextual information (such as authors, target area and document’s purpose). Also, information based on previous research on addressing socioeconomic inequalities in avoidable emergency admissions, such as who stands to benefit, was extracted [43]. Additionally, all documents were summarised according to a template designed according to the research’s aims. Data extraction and summaries were undertaken by L.M. and C.P.-C. A selection was doubled coded to enhance validity and any discrepancies were resolved by discussion.

### Analysis

Interviews and documents were coded and analysed independently based on a thematic analysis approach [45], managed by NVivo software. A combination of ‘interpretive’ and ‘positivist’ stance [34, 46] was taken which involved understanding meanings/contexts and processes as perceived from different perspectives (interviewees and documents). This allowed for an understanding of individual and shared social meanings/reasonings [34, 36]. For the documentary analysis, a combination of both content and thematic analysis as described by Bowen [47] informed by Braun and Clarke’s approach to thematic analysis [45] was used. This type of content analysis does not include the typical quantification but rather a review of the document for pertinent and meaningful passages of text/other data [47]. Both an inductive and deductive approach for the documentary analysis’ coding [46, 47] was chosen. The inductive approach was developed a posteriori; the deductive codes being informed by the interviews and previous findings from research addressing socioeconomic inequalities in avoidable emergency admissions [43]. In line with qualitative epistemological approach to enquiry, the interview and documentary findings were viewed as ‘truths’ in themselves with the acceptance that multiple realities can co-exist [48]. The analysis of each set of themes (with subthemes) from the documentary analysis and interviews were cross-referenced and integrated with each other to provide a cohesive in-depth analysis [49] by generating thematic maps to explore the relationships between the themes. The codes, themes and thematic maps were peer-reviewed continually with regular meetings between L.M., C.P.-C., J.L. and S.S. Direct quotes are provided from the interviews and documentary analysis. Some quotes from the documents are paraphrased to protect anonymity of the case study after following a set process considering a range of options. This involved searching each quote from the documentary analysis in Google and if the quote was found in the first page of the result, we shortened extracts and repeated the process. Where the shortened extracts were still identifiable, we were required to paraphrase that quote. Each paraphrased quote and original was shared and agreed with all the authors reducing the likelihood of inadvertently misinterpreting or misquoting. Where multiple components over large bodies of text were present in the documents, models were used to evidence the broadness, for example, using Dahlgren’s and Whitehead’s model of health determinants [1]. Due to the nature of the study, transcripts and findings were not shared with participants for checking but will be shared in a dissemination workshop in 2024.

### Patient and public involvement and engagement

Four public contributors from the National Institute for Health and Care Research (NIHR) Research Design Service (RDS) North East and North Cumbria (NENC) Public and Patient Involvement (PPI) panel have been actively engaged in this research from its inception. They have been part of the research advisory group along with professional stakeholders and were involved in the identification of the sampling frame’s key criteria. Furthermore, a diverse group of public contributors has been actively involved in other parts of the project including developing the moral argument around action by producing a public facing resource exploring what health inequalities mean to people and public views of possible solutions [50].

## Results

### Semi-structured interviews: description

Sixteen participants working in health or social care, identified through the documentary analysis or snowballing, were contacted for interview; fourteen consented to participate. No further interviews were sought as data sufficiency was reached whereby no new information or themes were being identified. Participant roles were broken down by NHS (*n* = 5), local authority/council (*n* = 8), and voluntary, community and social enterprise (VSCE) (*n* = 1). To protect the participants’ anonymity, their employment titles/status are not disclosed. However, a broad spectrum of interviewees with varying roles from senior health system leadership (including strategic and commissioner roles) to roles within provider organisations and the VSCE sector were included.

### Documentary analysis: description

75 documents were reviewed with documents considering regional (*n* = 20), local (*n* = 64) or neighbourhood (*n* = 2) area with some documents covering two or more areas. Table 2 summarises the respective number of each document type which included statutory documents to websites from across the system (NHS, local government and VSCE). 45 documents were named by interviewees and 42 documents were identified as either a core document or through snowballing from other documents. Of these, 12 documents were identified from both. The timescales of the documents varied and where possible to identify, was from 2014 to 2031.

**Table 2:** Number and types of documents

Type of document	Number^a^
Website/webpage	37
Strategy/plan	20
Annual report/report/evaluation	14
Framework	2
Audit	1
Joint Strategic Needs Assessment	1
Newsletter	1
PowerPoint presentation	1
Terms of Reference (TOR)	1
Toolkit	1

^a^Numbers may overlap as could be more than one type of document

### Integrative analysis of the documentary analysis and interviews

The overarching themes encompass:

- Understanding the local context
- Facilitators to tacking health inequalities: the assets
- Emerging risks and concerns

**Fig. 1:**
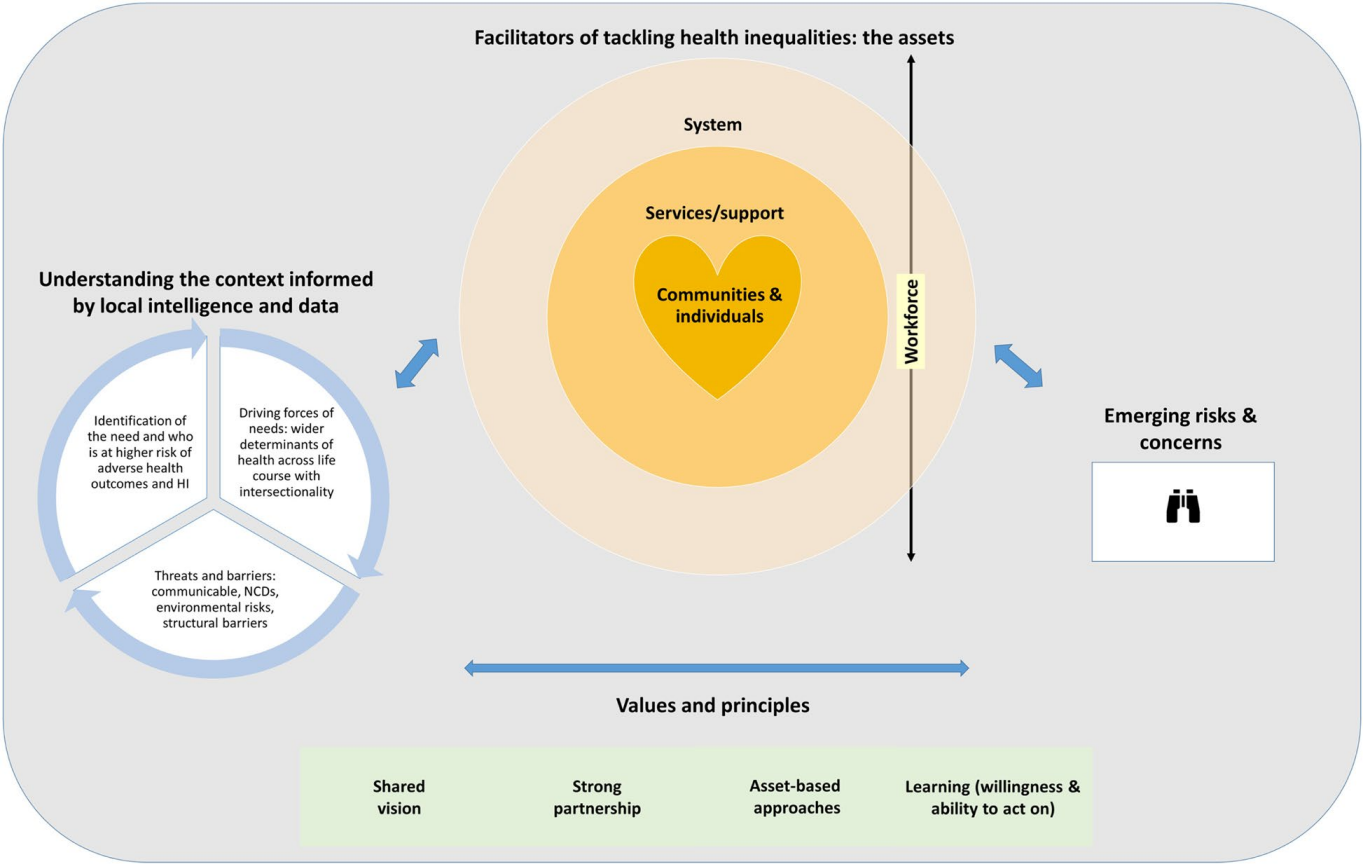
Diagram of the relationship between the key themes identified regarding tackling health inequalities and improving health in a local area informed by 2 previous work [14, 51]. NCDs = non-communicable diseases; HI = health inequalities

Figure 1 demonstrates the relationships between the main themes identified from the analysis for tackling health inequalities and improving health in this case study.

#### Understanding the local context

Understanding the local context was discussed extensively in both the documents and the interviews. This was informed by local intelligence and data that was routinely collected, monitored, and analysed to help understand the local context and where inequalities lie. More bespoke, in-depth collection and analysis were also described to get a better understanding of the situation. This not only took the form of quantitative but also considered qualitative data with lived experience:

*‛So, our data comes from going out to talk to people. I mean, yes, especially the voice of inequalities, those traditional mechanisms, like surveys, don't really work. And it's about going out to communities, linking in with third sector organisations, going out to communities, and just going out to listen…I think the more we can bring out those real stories. I mean, we find quotes really, really powerful in terms of helping people understand what it is that matters.’* (LP16).

However, there were limitations to the available data including the quality as well as having enough time to do the analysis justice. This resulted in difficulties in being able to fully understand the context to help identify and act on the required improvements.

*‘A lack of available data means we cannot quantify the total number of vulnerable migrants in [region]’* (Document V).

*‛So there’s lots of data. The issue is joining that data up and analysing it, and making sense of it. That’s where we don’t have the capacity.’* (LP15).

Despite the caveats, understanding the context and its data limitations were important to inform local priorities and approaches on tackling health inequalities. This understanding was underpinned by three subthemes which were understanding:

1. the population’s needs including identification of people at higher risk of worse health and health inequalities
2. the driving forces of those needs with acknowledgement of the impact of the wider determinants of health
3. the threats and barriers to physical and mental health, as well as wellbeing

Firstly, the population’s needs, including identification of people at higher risk of worse health and health inequalities, was important. This included considering risk factors, such as smoking, specific groups of people and who was presenting with which conditions. Between the interviews and documents, variation was seen between groups deemed at-risk or high-risk with the documents identifying a wider range. The groups identified across both included marginalised communities, such as ethnic minority groups, gypsy and travellers, refugees and asylum seekers as well as people/children living in disadvantaged area.

*‘There are significant health inequalities in children with asthma between deprived and more affluent areas, and this is reflected in A&E admissions.'* (Document J).

Secondly, the driving forces of those needs with acknowledgement of the impact of the wider determinants of health were described. These forces mapped onto Dahlgren’s and Whitehead’s model of health determinants [1] consisting of individual lifestyle factors, social and community networks, living and working conditions (which include access to health care services) as well as general socio-economic, cultural and environmental conditions across the life course.

*…. at the centre of our approach considering the requirements to improve the health and wellbeing of our area are the wider determinants of health and wellbeing, acknowledging how factors, such as housing, education, the environment and economy, impact on health outcomes and wellbeing over people’s lifetime and are therefore pivotal to our ambition to ameliorate the health of the poorest the quickest. (Paraphrased Document P).*

Thirdly, the threats and barriers to health included environmental risks, communicable diseases and associated challenges, non-communicable conditions and diseases, mental health as well as structural barriers. In terms of communicable diseases, COVID-19 predominated. The environmental risks included climate change and air pollution. Non-communicable diseases were considered as a substantial and increasing threat and encompassed a wide range of chronic conditions such as diabetes, and obesity.

*‛Long term conditions are the leading causes of death and disability in [case study] and account for most of our health and care spending. Cases of cancer, diabetes, respiratory disease, dementia and cardiovascular disease will increase as the population of [case study] grows and ages.’* (Document A).

Structural barriers to accessing and using support and/or services for health and wellbeing were identified. These barriers included how the services are set up, such as some GP practices asking for proof of a fixed address or form of identification to register. For example:

*Complicated systems (such as having to make multiple calls, the need to speak to many people/gatekeepers or to call at specific time) can be a massive barrier to accessing healthcare and appointments. This is the case particularly for people who have complex mental health needs or chaotic/destabilized circumstances. People who do not have stable housing face difficulties in registering for GP and other services that require an address or rely on post to communicate appointments.* (Paraphrased Document R).

A structural threat regarding support and/or services for health and wellbeing was the sustainability of current funding with future uncertainty posing potential threats to the delivery of current services. This also affected the ability to adapt and develop the services, or indeed build new ones.

*‛I would say the other thing is I have a beef [sic] [disagreement] with pilot studies or new innovations. Often soft funded, temporary funded, charity funded, partnership work run by enthusiasts. Me, I've done them, or supported people doing many of these. And they're great. They can make a huge impact on the individuals involved on that local area. You can see fantastic work. You get inspired and you want to stand up in a crowd and go, “Wahey, isn't this fantastic?” But actually the sad part of it is on these things, I've seen so many where we then see some good, positive work being done, but we can't make it permanent or we can't spread it because there's no funding behind it.’* (LP8).

#### Facilitators to tackling health inequalities: the assets

The facilitators for improving health and wellbeing and tackling health inequalities are considered as assets which were underpinned by values and principles.

#### Values driven supported by four key principles

Being values driven was an important concept and considered as the underpinning attitudes or beliefs that guide decision making [52]. Particularly, the system’s approach was underpinned by a culture and a system's commitment to tackle health inequalities across the documents and interviews. This was also demonstrated by how passionately and emotively some interviewees spoke about their work.

*‛There's a really strong desire and ethos around understanding that we will only ever solve these problems as a system, not by individual organisations or even just part of the system working together. And that feels great.’* (LP3).

Other values driving the approach included accountability, justice, and equity. Reducing health inequalities and improving health were considered to be the right things to do. For example:

*We feel strongly about social justice and being inclusive, wishing to reflect the diversity of [case study]. We campaign on subjects that are important to people who are older with respect and kindness.* (Paraphrased Document O).

Four key principles were identified that crosscut the assets which were:

- Shared vision
- Strong partnership
- Asset-based approaches
- Willingness and ability to act on learning

The mandated strategy, identifying priorities for health and wellbeing for the local population with the required actions, provided the shared vision across each part of the system, and provided the foundations for the work. This shared vision was repeated consistently in the documents and interviews from across the system.

*[Case study] will be a place where individuals who have the lowest socioeconomic status will ameliorate their health the quickest. [Case study] will be a place for good health and compassion for all people, regardless of their age.* (Paraphrased Document A).

*‛One thing that is obviously becoming stronger and stronger is the focus on health inequalities within all of that, and making sure that we are helping people and provide support to people with the poorest health as fast as possible, so that agenda hasn’t shifted.’* (LP7).

This drive to embed the reduction of health inequalities was supported by clear new national guidance encapsulated by the NHS Core20PLUS5 priorities. Core20PLUS5 is the UK's approach to support a system to improve their healthcare inequalities [53]. Additionally, the system's restructuring from Clinical Commissioning Groups (CCGs) to Integrated Care Boards (ICBs) and formalisation of the now statutory Integrated Care Systems (ICS) in England was also reported to facilitate the driving of further improvement in health inequalities. These changes at a regional and local level helped bring key partners across the system (NHS and local government among others) to build upon their collective responsibility for improving health and reducing health inequalities for their area [54].

*‛I don’t remember the last time we’ve had that so clear, or the last time that health inequalities has had such a prominent place, both in the NHS planning guidance or in the NHS contract.*’ (LP15).

*‛The Health and Care Act has now got a, kind of, pillar around health inequalities, the new establishment of ICPs and ICBs, and also the planning guidance this year had a very clear element on health inequalities.’* (LP12)

A strong partnership and collaborative team approach across the system underpinned the work from the documents and included the reoccurrence of the concept that this case study acted as one team: ‘Team [case study]'.

*Supporting one another to ensure [case study] is the best it can be: Team [case study]. It involves learning, sharing ideas as well as organisations sharing assets and resources, authentic partnerships, and striving for collective impact (environmental and social) to work towards shared goals*. (Paraphrased Document B).

This was corroborated in the interviews as working in partnership to tackle health inequalities was considered by the interviewees as moving in the right direction. There were reports that the relationship between local government, health care and the third sector had improved in recent years which was still an ongoing priority:

*‘I think the only improvement I would cite, which is not an improvement in terms of health outcomes, but in terms of how we work across [case study] together has moved on quite a lot, in terms of teams leads and talking across us, and how we join up on things, rather than see ourselves all as separate bodies' (LP15).*

*‘I think the relationship between local authorities and health and the third sector, actually, has much more parity and esteem than it had before.' (LP11)*

The approaches described above were supported by all health and care partners signing up to principles around partnership; it is likely this has helped foster the case study's approach. This also builds on the asset-based approaches that were another key principle building on co-production and co-creation which is described below.

***We begin with people****: instead of doing things to people or for them, we work with them, augmenting the skills, assets and strength of [case study]’s people, workforce and carers. ****We achieve****: actions are focused on over words and by using intelligence, every action hones in on the actual difference that we will make to ameliorate outcomes, quality and spend [case study]’s money wisely; ****We are Team [case study****]: having kindness, working as one organisation, taking responsibility collectively and delivering on what we agreed. Problems are discussed with a high challenge and high support attitude.* (Paraphrased Document D).

At times, the degree to which the asset-based approaches were embedded differed from the documents compared to the interviews, even when from the same part of the system. For example, the documents often referred to the asset-based approach as having occurred whilst interviewees viewed it more as a work in progress.

*‘We have re-designed many of our services to focus on needs-led, asset-based early intervention and prevention, and have given citizens more control over decisions that directly affect them*.’ (Document M).

*‘But we’re trying to take an asset-based approach, which is looking at the good stuff in communities as well. So the buildings, the green space, the services, but then also the social capital stuff that happens under the radar.’* (LP11).

A willingness to learn and put in action plans to address the learning were present. This enables future proofing by building on what is already in place to build the capacity, capability and flexibility of the system. This was particularly important for developing the workforce as described below.

*‘So we’ve got a task and finish group set up, […] So this group shows good practice and is a space for people to discuss some of the challenges or to share what interventions they are doing around the table, and also look at what other opportunities that they have within a region or that we could build upon and share and scale.’* (LP12).

These assets that are considered as facilitators are divided into four key levels which are the system, services and support, communities and individuals, and workforce which are discussed in turn below.

#### System

Firstly, the system within this case study was made up of many organisations and partnerships within the NHS, local government, VSCE sector and communities. The interviewees reported the presence of a strong VCSE sector which had been facilitated by the local council's commitment to funding this sector:

*‘Within [case study], we have a brilliant third sector, the council has been longstanding funders of infrastructure in [case study], third sector infrastructure, to enable those links [of community engagement] to be made'* (LP16).

In both the documents and interviews, a strong coherent strategic integrated population health management plan with a system’s approach to embed the reduction of health inequalities was evident. For example, on a system level regionally:

*‘To contribute towards a reduction in health inequalities we will: take a system wide approach for improving outcomes for specific groups known to be affected by health inequalities, starting with those living in our most deprived communities….’* (Document H).

This case study’s approach within the system included using creative solutions and harnessing technology. This included making bold and inventive changes to improve how the city and the system linked up and worked together to improve health. For example, regeneration work within the city to ameliorate and transform healthcare facilities as well as certain neighbourhoods *by having new green spaces, better transport links in order to improve city-wide innovation and collaboration* (paraphrased Document F) were described. The changes were not only related to physical aspects of the city but also aimed at how the city digitally linked up. Being a leader in digital innovation to optimise the health benefits from technology and information was identified in several documents.

‘*Having the best connected city using digital technology to improve health and wellbeing in innovative ways.’* (Document G).

The digital approaches included ongoing development of a digitalised personalised care record facilitating access to the most up-to-date information to developing as well as having the ‘*latest, cutting edge technologies’ (*Document *F)* in hospital care. However, the importance of not leaving people behind by embedding digital alternatives was recognised in both the documents and interviews.

‘*We are trying to just embed the culture of doing an equity health impact assessment whenever you are bringing in a digital solution or a digital pathway, and that there is always an alternative there for people who don’t have the capability or capacity to use it.*’ (LP1).

*The successful one hundred percent [redacted] programme is targeting some of our most digitally excluded citizens in [case study]. For our city to continue to thrive, we all need the appropriate skills, technology and support to get the most out of being online.* (Paraphrased Document Q)

This all links in with the system that functions in a ‘place' which includes the importance of where people are born, grow, work and live. Working towards this place being welcoming and appealing was described both regionally and locally. This included aiming to make the case study the place of choice for people.

*‘Making [case study] a centre for good growth becoming the place of choice in the UK to live, to study, for businesses to invest in, for people to come and work.’ *(Document G).

#### Services and support

Secondly, a variety of available services and support were described from the local authority, NHS, and voluntary community sectors. Specific areas of work, such as local initiatives (including targeted work or campaigns for specific groups or specific health conditions) as well as parts of the system working together with communities collaboratively, were identified. This included a wide range of work being done such as avoiding delayed discharges or re-admissions, providing high quality affordable housing as well as services offering peer support.

*‘We have a community health development programme called [redacted], that works with particular groups in deprived communities and ethnically diverse communities to work in a very trusted and culturally appropriate way on the things that they want to get involved with to support their health.’ (LP3*).

It is worth noting that reducing health inequalities in avoidable admissions was not often explicitly specified in the documents or interviews. However, either specified or otherwise inferred, preventing ill health and improving access, experience, and outcomes were vital components to addressing inequalities. This was approached by working with communities to deliver services in communities that worked for all people. Having co-designed, accessible, equitable integrated services and support appeared to be key.

*‘Reducing inequalities in unplanned admissions for conditions that could be cared for in the community and access to planned hospital care is key.’* (Document H)

*Creating plans with people: understanding the needs of local population and designing joined-up services around these needs.* (Paraphrased Document A).

‘*So I think a core element is engagement with your population, so that ownership and that co-production, if you're going to make an intervention, don't do it without because you might miss the mark.*’ (LP8).

Clear, consistent and appropriate communication that was trusted was considered important to improve health and wellbeing as well as to tackle health inequalities. For example, trusted community members being engaged to speak on the behalf of the service providers:

*‘The messenger is more important than the message, sometimes.’* (LP11).

This included making sure the processes are in place so that the information is accessible for all, including people who have additional communication needs. This was considered as a work in progress in this case study.

*‘I think for me, things do come down to those core things, of health, literacy, that digital exclusion and understanding the wider complexities of people.’ (LP12)*

‘*But even more confusing if you've got an additional communication need. And we've done quite a lot of work around the accessible information standard which sounds quite dry, and doesn't sound very- but actually, it's fundamental in accessing health and care. And that is, that all health and care organisations should record your communication preferences. So, if I've got a learning disability, people should know. If I've got a hearing impairment, people should know. But the systems don’t record it, so blind people are getting sent letters for appointments, or if I've got hearing loss, the right provisions are not made for appointments. So, actually, we're putting up barriers before people even come in, or can even get access to services.’* (LP16).

Flexible, empowering, holistic care and support that was person-centric was more apparent in the documents than the interviews.

*At the centre of our vision is having more people benefiting from the life chances currently enjoyed by the few to make [case study] a more equal place. Therefore, we accentuate the importance of good health, the requirement to boost resilience, and focus on prevention as a way of enabling higher quality service provision that is person-centred.* [Paraphrased Document N).

*Through this [work], we will give all children and young people in [case study], particularly if they are vulnerable and/or disadvantaged, a start in life that is empowering and enable them to flourish in a compassionate and lively city.* [Paraphrased Document M].

#### Communities and individuals

Thirdly, having communities and individuals at the heart of the work appeared essential and viewed as crucial to nurture in this case study. The interconnectedness of the place, communities and individuals were considered a key part of the foundations for good health and wellbeing.

*In [case study], our belief is that our people are our greatest strength and our most important asset. Wellbeing starts with people: our connections with our friends, family, and colleagues, our behaviour, understanding, and support for one another, as well as the environment we build to live in together*. (Paraphrased Document A).

A recognition of the power of communities and individuals with the requirement to support that key principle of a strength-based approach was found. This involved close working with communities to help identify what was important, what was needed and what interventions would work. This could then lead to improved resilience and cohesion.

*‛You can't make effective health and care decisions without having the voice of people at the centre of that. It just won't work. You won't make the right decisions.’* (LP16).

*‘Build on the strengths in ourselves, our families, carers and our community; working with people, actively listening to what matters most to people, with a focus on what’s strong rather than what’s wrong’* (Document G).

*Meaningful engagement with communities as well as strengths and asset-based approaches to ensure self-sufficiency and sustainability of communities can help communities flourish. This includes promoting friendships, building community resilience and capacity, and inspiring residents to find solutions to change the things they feel needs altering in their community*. (Paraphrased Document B).

This close community engagement had been reported to foster trust and to lead to improvements in health.

*‘But where a system or an area has done a lot of community engagement, worked really closely with the community, gained their trust and built a programme around them rather than just said, “Here it is. You need to come and use it now,” you can tell that has had the impact.*' (LP1).

#### Workforce

Finally, workforce was another key asset; the documents raised the concept of one workforce across health and care. The key principles of having a shared vision, asset-based approaches and strong partnership were also present in this example:

*By working together, the Health and Care sector makes [case study] the best area to not only work but also train for people of all ages. Opportunities for skills and jobs are provided with recruitment and engagement from our most disadvantaged communities, galvanizing the future’s health and care workforce. By doing this, we have a very skilled and diverse workforce we need to work with our people now as well as in the future.* (Paraphrased Document E).

An action identified for the health and care system to address health inequalities in case study 1 was ‘*the importance of having an inclusive workforce trained in person-centred working practices*’ (Document R). Several ways were found to improve and support workforce skills development and embed awareness of health inequalities in practice and training. Various initiatives were available such as an interactive health inequalities toolkit, theme-related fellowships, platforms and networks to share learning and develop skills.

*‛We've recently launched a [redacted] Fellowship across [case study’s region], and we've got a number of clinicians and managers on that………. We've got training modules that we've put on across [case study’s region], as well for health inequalities…we've got learning and web resources where we share good practice from across the system, so that is our [redacted] Academy.’* (LP2).

This case study also recognised the importance of considering the welfare of the workforce; being skilled was not enough. This had been recognised pre-pandemic but was seen as even more important post COVID-19 due to the impact that COVID-19 had on staff, particularly in health and social care.

*‛The impacts of the pandemic cannot be underestimated; our colleagues and services are fatigued and still dealing with the pressures. This context makes it even more essential that we share the responsibility, learn from each other at least and collaborate with each other at best, and hold each other up to be the best we can.’* (Document U).

#### Emerging risks and concerns

Concerns were raised such as the widening of health inequalities since the pandemic and cost of living crisis. Post-pandemic and Brexit, recruiting health, social care and third sector staff was compounding the capacity throughout this already heavily pressurised system.

*In [case study], we have seen the stalling of life expectancy and worsening of the health inequality gap, which is expected to be compounded by the effects of the pandemic.* (Paraphrased Document T)

*‘I think key barriers, just the immense pressure on the system still really […] under a significant workload, catching up on activity, catching up on NHS Health Checks, catching up on long-term condition reviews. There is a significant strain on the system still in terms of catching up. It has been really difficult because of the impact of COVID.’* (LP7).

*‘Workforce is a challenge, because the pipelines that we’ve got, we’ve got fewer people coming through many of them. And that’s not just particular to, I don't know, nursing, which is often talking talked [sic] about as a challenged area, isn't it? And of course, it is. But we’ve got similar challenges in social care, in third sector.’ (LP5).*

The pandemic was reported to have increased pressures on the NHS and services not only in relation to staff capacity but also regarding increases in referrals to services, such as mental health. Access to healthcare changed during the pandemic increasing barriers for some:

*‘I think people are just confused about where they're supposed to go, in terms of accessing health and care at the moment. It's really complex to understand where you're supposed to go, especially, at the moment, coming out of COVID, and the fact that GPs are not the accessible front door. You can't just walk into your GP anymore.’* (LP16).

*‘Meeting this increased demand [for work related to reducing ethnic inequalities in mental health] is starting to prove a challenge and necessitates some discussion about future resourcing.’* (Document S)

Several ways were identified to aid effective adaptation and/or mitigation. This included building resilience such as developing the existing capacity, capability and flexibility of the system by learning from previous work, adapting structures and strengthening workforce development. Considerations, such as a commitment to Marmot Principles and how funding could/would contribute, were also discussed.

*The funding’s [linked to Core20PLUS5] purpose is to help systems to ensure that health inequalities are not made worse when cost-savings or efficiencies are sought…The available data and insight are clear and [health inequalities are] likely to worsen in the short term, the delays generated by pandemic, the disproportionate effect of that on the most deprived and the worsening food and fuel poverty in all our places.* (Paraphrased Document L).

Learning from the pandemic was thought to be useful as some working practices had altered during COVID-19 for the better, such as needing to continue to embed how the system had collaborated and resist old patterns of working:

*‘So I think that emphasis between collaboration – extreme collaboration – which is what we did during COVID is great. I suppose the problem is, as we go back into trying to save money, we go back into our old ways of working, about working in silos. And I think we’ve got to be very mindful of that, and continue to work in a different way.’* (LP11).

Another area identified as requiring action, was the collection, analysis, sharing and use of data accessible by the whole system.

*‘So I think there is a lot of data out there. It’s just how do we present that in such a way that it’s accessible to everyone as well, because I think sometimes, what happens is that we have one group looking at data in one format, but then how do we cascade that out?’ (LP12)*

## Discussion

We aimed to explore a system’s level understanding of how a local area addresses health inequalities with a focus on avoidable emergency admissions using a case study approach. Therefore, the focus of our research was strategic and systematic approaches to inequalities reduction. Gaining an overview of what was occurring within a system is pertinent because local areas are required to have a regard to address health inequalities in their local areas [20, 21]. Through this exploration, we also developed an understanding of the system's processes reported to be required. For example, an area requiring action was viewed as the accessibility and analysis of data. The case study described having health inequalities ‘at the heart of its health and wellbeing strategy*’* which was echoed across the documents from multiple sectors across the system. Evidence of a values driven partnership with whole systems working was centred on the importance of place and involving people, with links to a ‘strong third sector*’*. Working together to support and strengthen local assets (the system, services/support, communities/individuals, and the workforce) were vital components. This suggested a system’s committed and integrated approach to improve population health and reduce health inequalities as well as concerted effort to increase system resilience. However, there was juxtaposition at times with what the documents contained versus what interviewees spoke about, for example, the degree to which asset-based approaches were embedded.

Furthermore, despite having *a priori* codes for the documentary analysis and including specific questions around work being undertaken to reduce health inequalities in avoidable admissions in the interviews with key systems leaders, this explicit link was still very much under-developed for this case study. For example, how to reduce health inequalities in avoidable emergency admissions was not often specified in the documents but could be inferred from existing work. This included work around improving COVID-19 vaccine uptake in groups who were identified as being at high-risk (such as older people and socially excluded populations) by using local intelligence to inform where to offer local outreach targeted pop-up clinics. This limited explicit action linking reduction of health inequalities in avoidable emergency admissions was echoed in the interviews and it became clear as we progressed through the research that a focus on reduction of health inequalities in avoidable hospital admissions at a systems level was not a dominant aspect of people’s work. Health inequalities were viewed as a key part of the work but not necessarily examined together with avoidable admissions. A strengthened will to take action is reported, particularly around reducing health inequalities, but there were limited examples of action to explicitly reduce health inequalities in avoidable admissions. This gap in the systems thinking is important to highlight. When it was explicitly linked, upstream strategies and thinking were acknowledged as requirements to reduce health inequalities in avoidable emergency admissions.

Similar to our findings, other research have also found networks to be considered as the system’s backbone [30] as well as the recognition that communities need to be central to public health approaches [51, 55, 56]. Furthermore, this study highlighted the importance of understanding the local context by using local routine and bespoke intelligence. It demonstrated that population-based approaches to reduce health inequalities are complex, multi-dimensional and interconnected. It is not about one part of the system but how the whole system interlinks. The interconnectedness and interdependence of the system (and the relevant players/stakeholders) have been reported by other research [30, 57], for example without effective exchange of knowledge and information, social networks and systems do not function optimally [30]. Previous research found that for systems to work effectively, management and transfer of knowledge needs to be collaborative [30], which was recognised in this case study as requiring action. By understanding the context, including the strengths and challenges, the support or action needed to overcome the barriers can be identified.

There are very limited number of case studies that explore health inequalities with a focus on hospital admissions. Of the existing research, only one part of the health system was considered with interviews looking at data trends [35]. To our knowledge, this research is the first to build on this evidence by encompassing the wider health system using wider-ranging interviews and documentary analysis. Ford et al. [35] found that geographical areas typically had plans to reduce total avoidable emergency admissions but not comprehensive plans to reduce health inequalities in avoidable emergency admissions. This approach may indeed widen health inequalities. Health inequalities have considerable health and costs impacts. Pertinently, the hospital care costs associated with socioeconomic inequalities being reported as £4.8 billion a year, mainly due to excess hospitalisations such as avoidable admissions [58] and the burden of disease lies disproportionately with our most disadvantaged communities, addressing inequalities in hospital pressures is required [25, 26].

### Implications for research and policy

Improvements to life expectancy have stalled in the UK with a widening of health inequalities [12]. Health inequalities are not inevitable; it is imperative that the health gap between the deprived and affluent areas is narrowed [12]. This research demonstrates the complexity and intertwining factors that are perceived to address health inequalities in an area. Despite the evidence of the cost (societal and individual) of avoidable admissions, explicit tackling of inequality in avoidable emergency admissions is not yet embedded into the system, therefore highlights an area for policy and action. This in-depth account and exploration of the characteristics of ‘whole systems’ working to address health inequalities, including where challenges remain, generated in this research will be instrumental for decision makers tasked with addressing health and care inequalities.

This research informs the next step of exploring each identified theme in more detail and moving beyond description to develop tools, using a suite of multidimensional and multidisciplinary methods, to investigate the effects of interventions on systems as previously highlighted by Rutter et al. [5].

### Strengths and limitations

Documentary analysis is often used in health policy research but poorly described [44]. Furthermore, Yin reports that case study research is often criticised for not adhering to ‘systematic procedures’ p. 18 [41]. A clear strength of this study was the clearly defined boundary (in time and space) case as well as following a defined systematic approach, with critical thought and rationale provided at each stage [34, 41]. A wide range and large number of documents were included as well as interviewees from across the system thereby resulting in a comprehensive case study. Integrating the analysis from two separate methodologies (interviews and documentary analysis), analysed separately before being combined, is also a strength to provide a coherent rich account [49]. We did not limit the reasons for hospital admission to enable a broad as possible perspective; this is likely to be a strength in this case study as this connection between health inequalities and avoidable hospital admissions was still infrequently made. However, for example, if a specific care pathway for a health condition had been highlighted by key informants this would have been explored.

Due to concerns about identifiability, we took several steps. These included providing a summary of the sectors that the interviewees and document were from but we were not able to specify which sectors each quote pertained. Additionally, some of the document quotes required paraphrasing. However, we followed a set process to ensure this was as rigorous as possible as described in the methods section. For example, where we were required to paraphrase, each paraphrased quote and original was shared and agreed with all the authors to reduce the likelihood to inadvertently misinterpreting or misquoting.

The themes are unlikely to represent an exhaustive list of the key elements requiring attention, but they represent the key themes that were identified using a robust methodological process. The results are from a single urban local authority with high levels of socioeconomic disadvantage in the North of England which may limit generalisability to different contexts. However, the findings are still generalisable to theoretical considerations [41]. Attempts to integrate a case study with a known framework can result in ‘force-fit’ [34] which we avoided by developing our own framework (Fig. 1) considering other existing models [14, 59]. The results are unable to establish causation, strength of association, or direction of influence [60] and disentangling conclusively what works versus what is thought to work is difficult. The documents’ contents may not represent exactly what occurs in reality, the degree to which plans are implemented or why variation may occur or how variation may affect what is found [43, 61]. Further research, such as participatory or non-participatory observation, could address this gap.

## Conclusions

This case study provides an in-depth exploration of how local areas are working to address health and care inequalities, with a focus on avoidable hospital admissions. Key elements of this system’s reported approach included fostering strategic coherence, cross-agency working, and community-asset based working. An area requiring action was viewed as the accessibility and analysis of data. Therefore, local areas could consider the challenges of data sharing across organisations as well as the organisational capacity and capability required to generate useful analysis in order to create meaningful insights to assist work to reduce health and care inequalities. This would lead to improved understanding of the context including where the key barriers lie for a local area. Addressing structural barriers and threats as well as supporting the training and wellbeing of the workforce are viewed as key to building resilience within a system to reduce health inequalities. Furthermore, more action is required to embed reducing health inequalities in avoidable admissions explicitly in local areas with inaction risking widening the health gap.

### Supplementary Information

Supplementary Material 1.

Supplementary Material 2.

## Data Availability

Individual participants’ data that underlie the results reported in this article and a data dictionary defining each field in the set are available to investigators whose proposed use of the data has been approved by an independent review committee for work. Proposals should be directed to weima@sdu.edu.cn to gain access, data requestors will need to sign a data access agreement. Such requests are decided on a case by case basis.
