# Supplementary material for: How can health systems approach reducing health inequalities? An in-depth qualitative case study in the UK
Source: BMC Public Health. 2024 Aug 10;24:2168. doi: 10.1186/s12889-024-19531-5 (PMC11316387; doi:10.1186/s12889-024-19531-5)
Supplement: Supplementary file 1 — Supplementary Material 1. [file 12889_2024_19531_MOESM1_ESM.zip › Interviewers.docx]

**Interviewers’ Details:** C.P.-C. (she/her) is a Public Health Registrar with 1-year qualitative experience; L. M. (she/her) is an experienced qualitative researcher with over 5 years’ experience. Both interviewers had limited experience working in the field of health inequalities research at the time of the interviews. L.M. organised all the interviews and had no prior relationship with the participants apart from email correspondence about the study.
