# Supplementary material for: How can health systems approach reducing health inequalities? An in-depth qualitative case study in the UK
Source: BMC Public Health. 2024 Aug 10;24:2168. doi: 10.1186/s12889-024-19531-5 (PMC11316387; doi:10.1186/s12889-024-19531-5)
Supplement: Supplementary file 1 — Supplementary Material 1. [file 12889_2024_19531_MOESM1_ESM.zip › Supplementary Data 1 Topic Guide for Interviews.docx]

Supplementary data 1: Topic Guide for Case Study Area 1

Introduction and Welcome

- Consent – e-consent returned?
- To the researcher
- Aims of the project
  - To explore and develop an understanding of how local areas address inequalities with a focus on avoidable emergency admissions.
  - To co-produce with patients, the public and health professionals, practical learning to help local area decision makers reduce inequalities.
- Aims of the interview
  - Get an overview of their role generally, then in relation to Health inequalities (HI) and avoidable emergency admissions (AEA) in particular
  - Identify key people in local area with a role in AEA
  - Identify key interventions around reducing HI and AEA

What is your role?

- Time in post
- Background

To what extent are HI/AEA a priority in your work

- Expand / give examples
- Collection of data?
  - Where is the data sourced?
  - Type of data
  - Is the data useful?
  - Enough evidence?
  - How is data used?
- Who do you link in with?
  - Meetings/minutes/reports?
  - Terms of reference for groups?
- What factors affect HI and AEA in your area?
- What HI/AEA work have you been involved with over the last 2-5 years?

In terms of HI/AEA what are the key interventions/programmes in your area

- Who is involved?
  - Service users/patient/public voices?
  - How is information collected/collated?
  - Representative?
- Staff training?
- Any documents/ reports?
  - Can they screen share/put links in chat?
  - Can they share terms of reference?
  - Can they share reports/minutes of meetings?
- How successful have they been?
- What improvements / changes have you seen?
- What makes for successful interventions in this area?
- What are the key barriers?

Anything you want to tell me about HI and AEA in this area that we haven’t covered?

If time allows ask interviewee (if they haven’t already done so) to name/share their 3 contacts/groups and documents/websites etc. This can be in the form of screen sharing which will be captured in the recording or paste links in chat.
